# Inhibitory Effect of *Boesenbergia rotunda* and Its Major Flavonoids, Pinostrobin and Pinocembrin on Carbohydrate Digestive Enzymes and Intestinal Glucose Transport in Caco-2 Cells

**DOI:** 10.3390/ijms262010158

**Published:** 2025-10-19

**Authors:** Sathid Aimjongjun, Nopawit Khamto, Vanatsanan Buangamdee, Thanet Sornda, Jukkarin Srivilai, Nanteetip Limpeanchob

**Affiliations:** 1 Department of Biochemistry, Faculty of Medical Science, Naresuan University, Phitsanulok 65000, Thailand; sathida@nu.ac.th (S.A.);; 2 Center of Excellence in Medical Biotechnology, Faculty of Medical Science, Naresuan University, Phitsanulok 65000, Thailand; 3 Unit of Excellence for Sustainable Innovation in Cosmetics, Natural Products and Pharmaceuticals (SICNP), School of Pharmaceutical Sciences, University of Phayao, Phayao 56000, Thailand; 4 Pharmacology Research Unit, Department of Pharmacy Practice, Center of Excellence for Innovation in Chemistry, Faculty of Pharmaceutical Sciences, Naresuan University, Phitsanulok 65000, Thailand

**Keywords:** pinostrobin, pinocembrin, antidiabetic, α-glucosidase, glucose transporters

## Abstract

*Boesenbergia rotunda* (L.) Mansf., commonly known as fingerroot or “Kra-Chai,” is a traditional Thai medicinal plant used for treating digestive and metabolic disorders. Recent evidence highlights its potential role in controlling hyperglycemia, though its active compounds and mechanisms remain unclear. This study evaluated the antidiabetic activity of *B. rotunda* crude extract and its major flavonoids, pinostrobin and pinocembrin, through in vitro enzyme inhibition and cellular glucose transport assays. Pinocembrin exhibited the strongest inhibition of both α-amylase and α-glucosidase, while pinostrobin and the crude extract showed moderate effects. In Caco-2 cells, the crude extract reduced glucose uptake, whereas both flavonoids markedly inhibited transport under glucose-depleted conditions, suggesting interaction with sodium-dependent glucose transporters (SGLTs). Under high-glucose conditions, their effects were minimal, indicating limited activity on facilitative glucose transporters (GLUTs). Moreover, molecular docking studies revealed that pinostrobin and pinocembrin bind within the glucose transporter channels of SGLT1 and SGLT2, blocking glucose passage and supporting the experimental findings. Overall, *B. rotunda*, particularly pinocembrin, demonstrates notable in vitro antidiabetic potential through enzyme inhibition and SGLT modulation. Further in vivo investigations are warranted to validate its hypoglycemic properties and identify additional active compounds.

## 1. Introduction

Type II diabetes mellitus (T2DM) has emerged as one of the most pressing global health challenges worldwide [1]. T2DM, characterized by insulin resistance and relative insulin deficiency, accounts for approximately 90–95% of all diabetes cases [1]. The condition is closely associated with lifestyle factors such as poor diet, physical inactivity, and obesity, and the prevalence continues to rise, particularly with rapid economic development and urbanization [2]. A key contributor to the development and progression of T2DM is chronic hyperglycemia caused by various nutritional factors, such as the excessive intake of carbohydrates, especially refined sugar and simple saccharides [3]. Unlike complex carbohydrates or fiber-rich foods, simple sugars are rapidly absorbed, causing sharp spikes in blood glucose levels and placing chronic stress on insulin production and function. Over time, this contributes to insulin resistance [4]. To prevent this disease, dietary intake strategies that modulate carbohydrate digestion and glucose absorption have garnered significant attention for their potential to preclude or delay the onset of the disease. In particular, inhibiting the enzymatic activity of α-amylase and α-glucosidase enzymes responsible for breaking down complex carbohydrates into absorbable sugars can slow glucose release into the bloodstream [5]. Additionally, moderate suppression of the sodium-glucose cotransporter1 (SGLT1), which facilitates intestinal glucose uptake, may further aid in controlling postprandial blood glucose spikes [6,7]. Natural food components such as polyphenols, flavonoids, and certain dietary fibers have demonstrated such effects, positioning functional foods as a promising tool in the dietary management and prevention of T2DM [8,9].

*Boesenbergia rotunda* (Linn.) Mansf, commonly known as fingerroot, is a medicinal plant traditionally and widely used in Southeast Asia and highly valued for its potent and diverse therapeutic properties. Among its bioactive constituents, the flavonoids pinostrobin and pinocembrin, which are among the major bioactive compounds, have recently attracted considerable scientific attention for their potential antidiabetic effects [10]. Research indicates that pinocembrin not only exhibits significant α-glucosidase inhibitory activity against maltase and sucrase but also inhibits pancreatic amylase enzyme activity, suggesting its role in modulating postprandial hyperglycemia by delaying carbohydrate digestion [11]. Additionally, pinocembrin has demonstrated the ability to inhibit the formation of advanced glycation end-products (AGEs), which are implicated in the progression of diabetic complications [11]. Pinostrobin, on the other hand, has shown anti-inflammatory and antioxidant properties, which may contribute to its protective effects against diabetes-induced oxidative stress and inflammation [10]. Additionally, pinostrobin and pinocembrin have attracted significant interest due to their roles in modulating glucose metabolism. Recent studies suggest that these compounds may influence glucose homeostasis not only by inhibiting carbohydrate-digesting enzymes like α-glucosidase and α-amylase but also by regulating the activity or expression of glucose transporters such as GLUT4 and SGLT1 [11]. Notably, pinocembrin has been shown to exert a markedly selective and potentially inhibitory effect on SGLT1-mediated glucose uptake [12,13] while also possibly enhancing GLUT4 translocation in a context-dependent and pathway-specific manner and promoting increased cellular glucose uptake, which is a key mechanism in improving insulin sensitivity. Meanwhile, antioxidant and anti-inflammatory activities of pinostrobin may indirectly support glucose transporter function by reducing oxidative stress, which impairs insulin signaling pathways.

Although many studies have demonstrated the antidiabetic properties of pinostrobin and pinocembrin, identifying them as effective anti-glycation agents, as well as α-amylase and α-glucosidase inhibitors, their precise molecular mechanisms remain insufficiently explored. These findings suggest their potential role in modulating postprandial glucose levels. However, research on *B. rotunda* extracts and their bioactive flavonoids is still in the early stage, particularly concerning their molecular interactions and functional mechanisms. To date, no studies have specifically examined the direct effects of pinostrobin or pinocembrin on membrane-bound α-glucosidase activity, which mimics the enzyme’s physiological function in the human small intestine, providing a relevant in vitro model for assessing intestinal glucose digestion and absorption. by GLUT- and SGLT1-mediated glucose uptake in Caco-2 cell models. So, the present study demonstrates the hypoglycemic activity of pinostrobin and pinocembrin through in vitro assays targeting Caco-2 cells’ intestinal membrane-bound enzymes and glucose transporters. Additionally, computational modeling was employed to predict the molecular interactions of these compounds with human target enzymes and transporters. These findings collectively support the comprehensive antidiabetic potential of *B*. *rotunda*-derived compounds, pinostrobin and pinocembrin.

## 2. Results

### 2.1. Plant Extraction and Isolation

**Figure 1:**
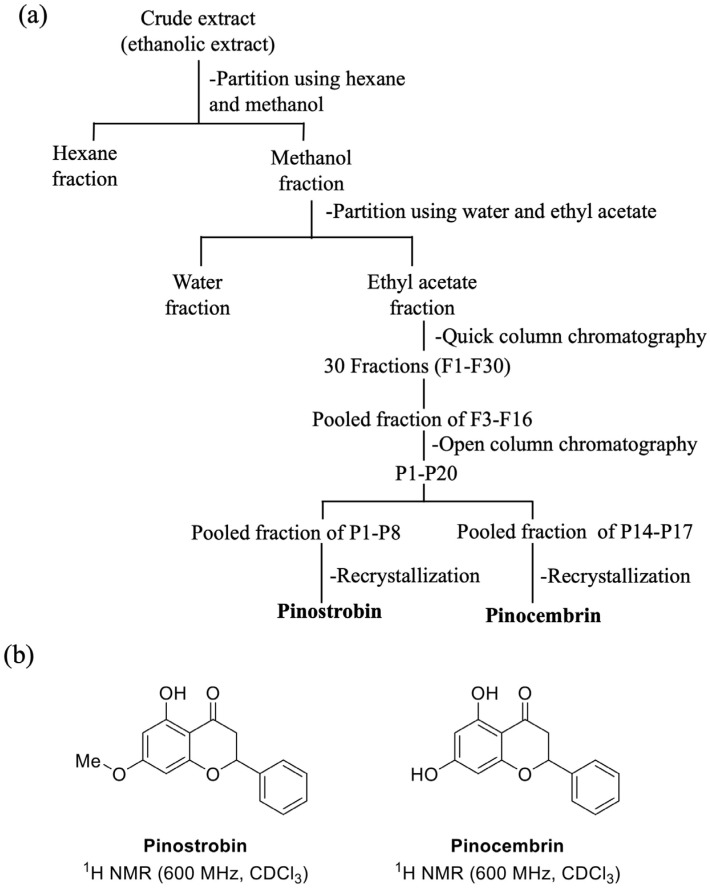
(**a**) Scheme of pinostrobin and pinocembrin isolation; (**b**) molecular structure of pinostrobin and pinocembrin.

The extraction of *B*. *rotunda* using ethanol solvent provided a % yield of 12% (36 g) of crude ethanolic extract. After partitioning to obtain the enriched fraction by hexane, a process yield of 30% was achieved; the main fraction was found to be significant in the ethyl acetate fraction, with 68%, and a small amount of yield was found in the water fraction, with only 16.8%. After fractionation using chromatography and crystallization techniques (Figure 1a), crystals of pinocembrin were obtained, with a yield of 0.24%, and they had an Rf value of 0.3 (hexane–ethyl acetate (8:2)), *m*/*z* 255.0660 [M + H]^+^, UV λmax 289 nm; ^1^H-NMR results are shown in Table 1 and Figure 1b. Pinostrobin was isolated and exhibited a % yield of 5.76; its ^1^H-NMR data are shown in Table 1 and Figure 1b.

### 2.2. Method Validation and Quantification of Major Bioactive Compounds by HPLC

**Figure 2:**
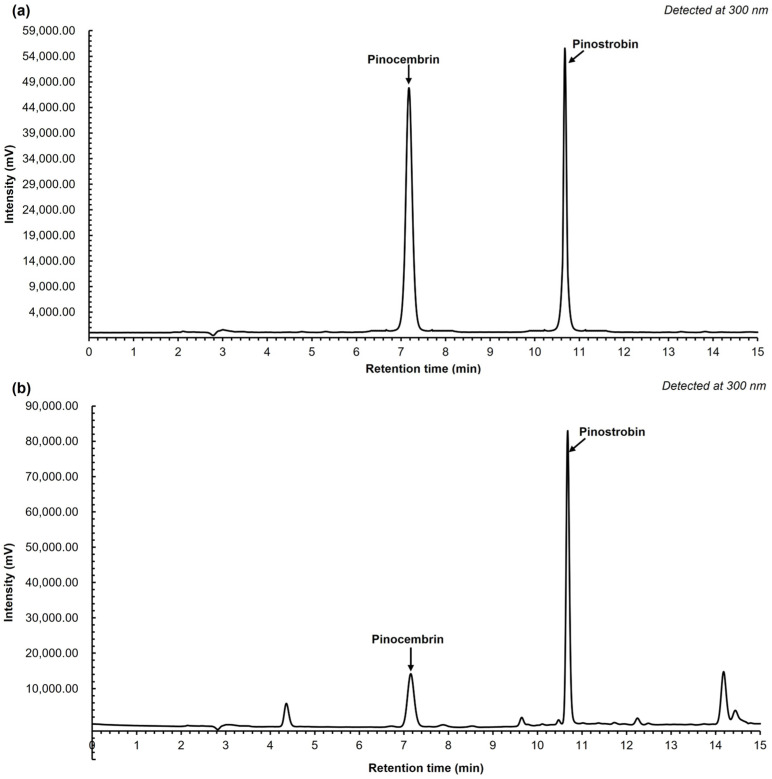
HPLC chromatogram of reference standard pinocembrin (7.17 min) and pinostrobin (10.67 min) at a concentration of 10 µg/ mL (**a**) and *B*. *routunda* extract at 1 mg/mL (**b**) under the detection at 300 nm.

The quantification of pinocembrin and pinostrobin was carried out using high-performance liquid chromatography (HPLC). Method specificity was confirmed by evaluating the resolution factor, which exceeded 1.5, indicating adequate separation of the two analytes. The method provided an analysis time of only 12 min (Figure 2) and another 3 min for a post-run. The reference standards of pinocembrin and pinostrobin exhibited retention times of 7.17 and 10.67 min, respectively. The ethyl acetate fraction showed identical retention times, confirming the presence of both compounds in the extract. Validation parameters are summarized in Table 2. Calibration curves for both pinocembrin and pinostrobin were established over the concentration range of 1–70 µg/mL, with linear regression equations displaying coefficients of determination (R^2^) ≥ 0.999. The limits of detection (LODs) for pinocembrin and pinostrobin were 0.11 and 0.046 µg/mL, respectively, while the limits of quantification (LOQs) were 0.30 and 0.15 µg/mL, reflecting sensitivity in the nano- to microgram range. Method robustness was assessed through intra- and inter-day precision, presenting %CV values below 1% (Table 1). Accuracy was confirmed by recovery studies, in which known amounts of pinocembrin and pinostrobin were spiked into the extract matrix. Recovery rates for both analytes ranged from 90% to 110%, indicating acceptable accuracy and precision. For extract standardization, the contents of pinostrobin and pinocembrin in the ethyl acetate fraction were quantified. The extract contained 21.4 ± 0.05% *w*/*w* of pinostrobin and 9.3 ± 0.02% *w*/*w* of pinocembrin.

### 2.3. Effects of Pinostrobin and Pinocembrin on α-Amylase Activity

**Figure 3:**
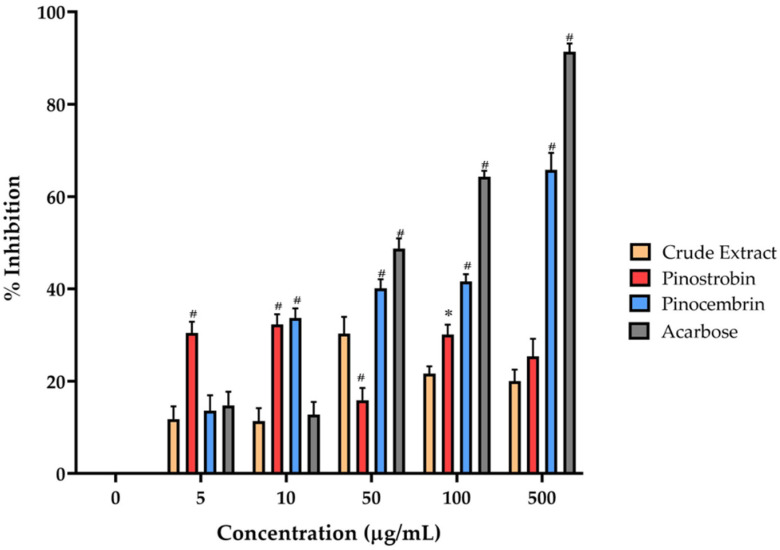
The alpha-amylase inhibitory activity of pinostrobin, pinocembrin, and the crude extract concentrations of 5, 10, 50, 100, and 500 µg/mL. Acarbose at the same concentration was used as a positive control. Bars marked with different symbols are significantly different compared to the crude extract (* *p* < 0.05, # *p* < 0.001).

Pinostrobin, pinocembrin, and the crude extract of *B. rotunda* were evaluated for their α-amylase inhibitory activity, which may suggest potential antidiabetic effects. The percentage inhibition for each compound is presented in Figure 3, alongside the commercially available α-amylase and α-glucosidase inhibitor, acarbose, for comparison. Most compounds exhibited a concentration-dependent inhibitory effect, except for the crude extract and pinostrobin, which showed minimal activity. Pinocembrin demonstrated notable inhibition, reaching approximately 65% at a concentration of 500 µg/mL, while acarbose inhibited 93% of enzyme activity at the same concentration. Although pinostrobin and the crude extract displayed weak inhibition, pinocembrin exhibited significantly higher activity than the other test compounds, albeit still lower than that of acarbose.

### 2.4. Effects of Pinostrobin and Pinocembrin on α-Glucosidase Activity

**Figure 4:**
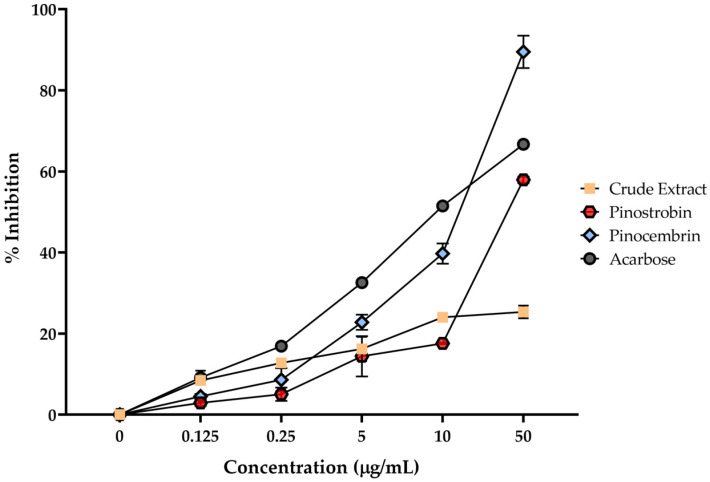
α-Glucosidase inhibitory activities of pinostrobin, pinocembrin, and the crude extract of *B*. *rotunda*, compared with the standard inhibitor acarbose. Various concentrations (0.125, 0.25, 5, 10, and 50 μg/mL) were used to evaluate the inhibitory effects of each compound. Acarbose (*p* < 0.001) at the same concentration.

The in vitro α-glucosidase inhibitory activity was assessed by measuring the release of *p*-nitrophenol from PNPG, a substrate cleaved by α-glucosidase enzymes that hydrolyze α(1→4) glycosidic bonds in disaccharides to produce monosaccharides. All tested compounds demonstrated α-glucosidase inhibitory activity in a dose-dependent manner, with more than 50% inhibition observed at the highest concentration tested (50 μg/mL), as shown in Figure 4. Among the compounds, pinocembrin exhibited the strongest inhibitory effect at the highest concentration, followed by acarbose (the positive control) and pinostrobin. The crude extract of *B*. *rotunda* showed the weakest activity compared to the isolated compounds.

### 2.5. Cytotoxicity of Pinostrobin and Pinocembrin on Caco-2 Cell

**Figure 5:**
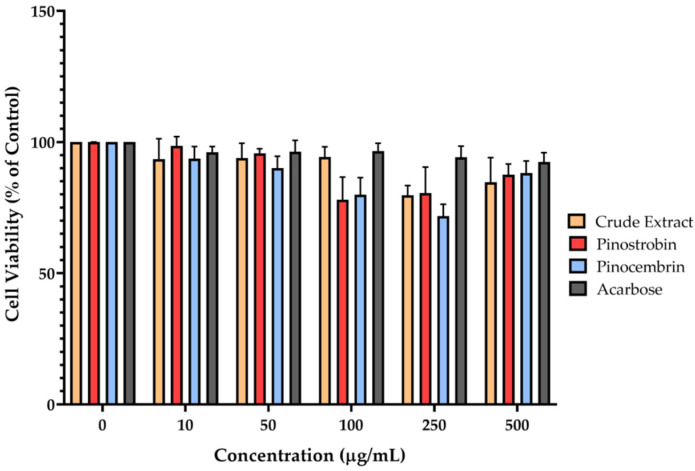
Cytotoxicity of pinostrobin, pinocembrin, and crude extract in Caco-2 cells. The cell viability was resolved using the MTT assay. After adhering the cells to the plate, the cells were treated with 10, 50, 100, 250, and 500 μg/mL of the compounds, and incubated for 24 h. The data are presented as mean ± SEM of the three experiments.

Mitochondrial enzyme activity, which reflects cell viability, was assessed using the MTT assay. This method relies on the reduction of the MTT reagent to insoluble formazan crystals by mitochondrial dehydrogenases in metabolically active cells. The results indicated that none of the tested compounds—pinostrobin, pinocembrin, or the crude extract of *B*. *rotunda*—exhibited significant cytotoxic effects on Caco-2 cells after 24 h of exposure (Figure 5). Cell viability remained above 80% across all tested concentrations, suggesting that the compounds are generally non-toxic at the doses evaluated.

### 2.6. Effects of Pinostrobin and Pinocembrin on Membrane-Bound α-Glucosidase Activity in Caco-2 Cells

**Figure 6:**
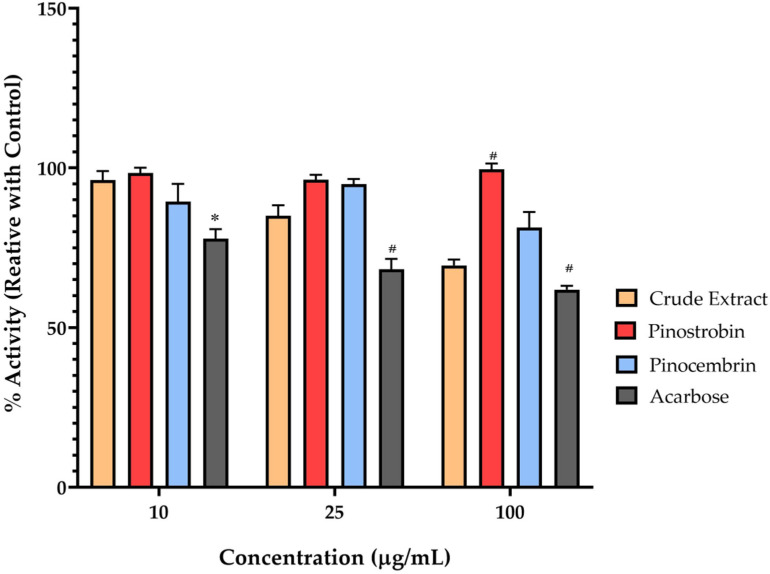
Percentage of maltase activity in membrane-bound maltase Caco-2 cells using maltose as the substrate. Cells were treated with acarbose, pinostrobin, pinocembrin, and crude extract. Data are presented as mean ± SEM. Specific enzyme activity was calculated, and relative activity was determined by comparison with the control group containing substrate and enzyme only. Bars labeled with symbol letters indicate statistically significant differences compared to no-compound-treated cells (* *p* < 0.05, # *p* < 0.001).

α-Glucosidase is a key membrane-bound enzyme located in the epithelial lining of the small intestine, responsible for hydrolyzing disaccharides into glucose, which is subsequently absorbed into the bloodstream. Therefore, inhibiting this enzyme is considered an effective approach for reducing postprandial blood glucose levels. In this study, the crude extract of *B*. *rotunda* demonstrated a concentration-dependent inhibition of disaccharidase (maltase) activity in Caco-2 cells. At concentrations of 10, 25, and 100 μg/mL, the residual maltase activity was approximately 95%, 80%, and 75%, respectively. These activities were expressed as a percentage of enzyme activity relative to the untreated control for comparison with the pharmaceutical standard, acarbose (Figure 6). Acarbose significantly reduced maltase activity by 25%, 30%, and 35% at the same respective concentrations. In contrast, pinostrobin and pinocembrin exhibited minimal inhibitory effects on maltase activity at all tested concentrations.

### 2.7. Effects of Pinostrobin and Pinocembrin on Glucose Uptake in Caco-2 Cells

Caco-2 cells are widely recognized as a reliable in vitro model for studying intestinal glucose uptake. As shown in Table 3, the crude extract of *B*. *rotunda* significantly reduced glucose uptake at concentrations of 100, 200, and 500 μg/mL, with corresponding uptake levels of 61.03%, 53.81%, and 66.58%, respectively. Among the tested compounds, Pinostrobin exhibited the most pronounced inhibitory effect on glucose uptake, significantly reducing 2-NBDG absorption to 49.97% and 50.51% at concentrations of 250 and 500 μg/mL, respectively. Pinocembrin also showed notable inhibition, with uptake activity reduced to 64.11% and 55.01% when compared to the same concentrations of pinostrobin. The positive control, phlorizin, known as a selective SLGT inhibitor, demonstrated strong inhibitory activity, reducing glucose uptake by 56.33% at 500 μg/mL. In contrast, the selective GLUT inhibitor, phloretin, showed only a mild effect on glucose uptake under the same conditions.

### 2.8. Effects of Pinostrobin and Pinocembrin on Transepithelial Glucose Transport in Caco-2 Cells

**Figure 7:**
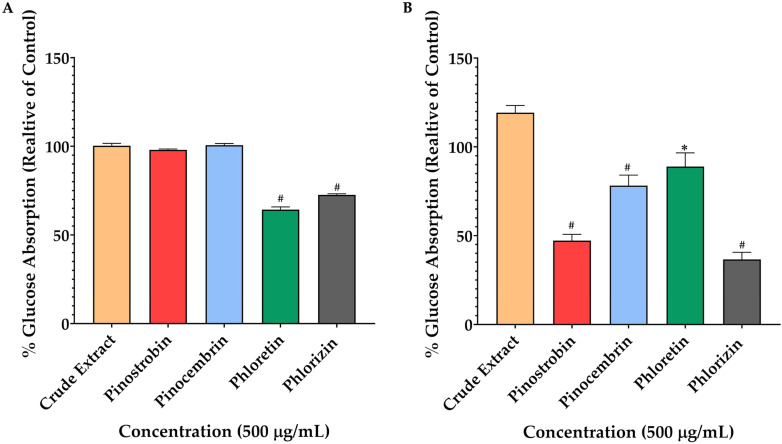
Effects of pinostrobin, pinocembrin, and crude extract on glucose transport across Caco-2 cell monolayers. Caco-2 cells were treated with 500 μg/mL of each compound, along with the SGLT1 inhibitor (phlorizin) and the GLUT inhibitor (phloretin), for 4 h at 37 °C. Glucose transport was assessed under two conditions: high-glucose medium, representing GLUT-mediated transport (**A**), and glucose-free medium, representing SGLT1-mediated transport (**B**). Bars labeled with different symbols indicate statistically significant differences to untreated cells (* *p* < 0.05, # *p* < 0.001).

To investigate the involvement of glucose transporters in the transcellular movement of glucose across the Caco-2 monolayer, the concentration of 2-NBDG on the basolateral side of the transwell system was measured. When cells were pretreated with phloretin and phlorizin under high-glucose conditions, a significant reduction in 2-NBDG transport was observed, indicating inhibition of glucose transporter activity. In contrast, no significant effect was detected with the crude extract, pinostrobin, or pinocembrin under the same conditions (Figure 7A). However, in glucose-free conditions, 2-NBDG transport was notably reduced by pinostrobin, pinocembrin, and the positive controls, phloretin and phlorizin (*p* < 0.01) (Figure 7B). Specifically, pinostrobin at 500 μg/mL showed a maximum reduction in absorption to 49%, followed by pinocembrin with a 75% uptake. Interestingly, the inhibitory effect of pinostrobin was comparable to that of phlorizin at the same concentration (45% reduction, *p* > 0.01). Among all tested compounds, phloretin exhibited the least inhibitory effect, with an uptake inhibition rate of 85% (*p* < 0.05) in glucose-free conditions.

### 2.9. Molecular Docking and Molecular Dynamics Simulations

The possible binding orientations of potent inhibitors, including phlorizin, phloretin, pinostrobin, and pinocembrin, within the binding sites of SGLT1 and SGLT2 were predicted using molecular docking. All inhibitors were found to bind effectively within the active sites of the respective receptors. The calculated binding energies (Table 1) indicated that phlorizin exhibited the strongest binding affinity across both targets, followed by phloretin.

**Figure 8:**
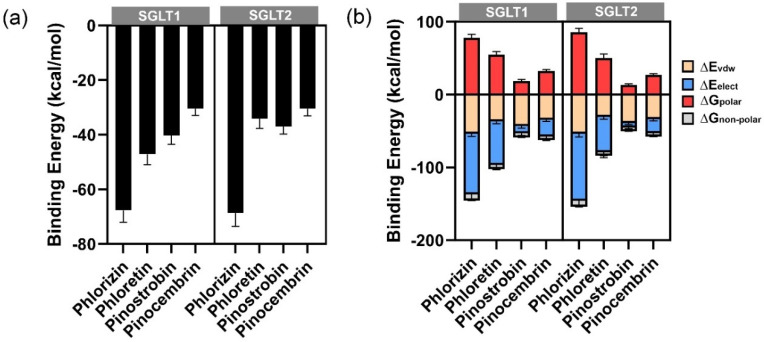
The MM-GBSA calculations of potent inhibitors with SGLT1 and SGLT2 receptors: (**a**) relative binding free energy between protein-ligand complexes; (**b**) energy components.

Molecular dynamics simulations were performed to gain insights into the stability and dynamic behavior of the protein-ligand complexes. Three distinct binding poses were selected for independent MD simulations to enhance conformational sampling. According to the RMSD of Cα of protein structure (Figure S1), the simulations showed that all complexes reached equilibrium during the simulation timescale. The complexes with the lowest binding energies were selected for visualization and further analysis (Figure S2). Relative binding free energies were calculated using the MM-GBSA method based on the MD trajectories (Table 4). The results confirmed that phlorizin consistently demonstrated the strongest binding affinity, particularly toward SGLT1 and SGLT2, aligning with its known role as a selective inhibitor for these transporters [14]. Among the other compounds, pinostrobin exhibited greater binding affinity than pinocembrin across all targets (Table 1 and Figure 8a).

The analysis of energy components (Figure 8b) revealed that all potent inhibitors exhibited similar van der Waals energy contributions. However, the electrostatic and polar solvation energies had the most significant influence on the overall binding energy. Phlorizin showed the strongest electrostatic interactions, attributed to its sugar moiety (glycone), which contributed to extensive hydrogen bonding. The strength of electrostatic interactions decreased with the reduction in the number of hydroxy groups, as observed in phloretin, pinostrobin, and pinocembrin.

**Figure 9:**
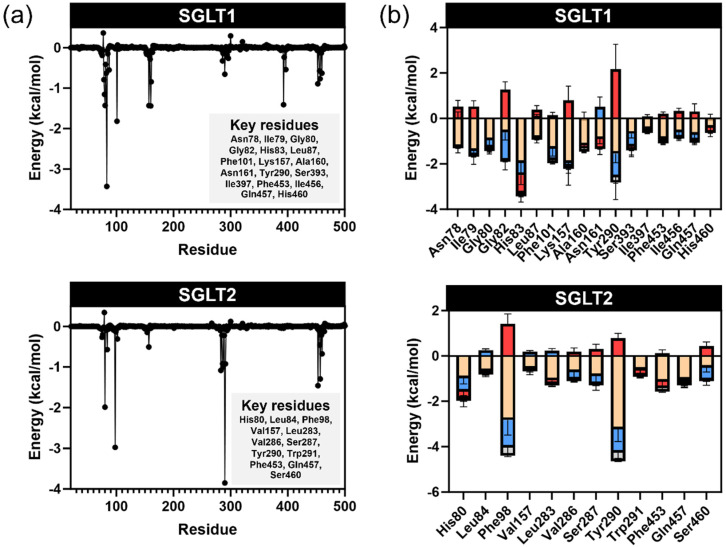
The MM-GBSA calculation results between pinostrobin and SGLTs: (**a**) per-residue energy decomposition analysis of MM-GBSA calculations. The black line indicates the total binding energy contribution of each residue.; (**b**) energy components of each key residue. The energy components are represented by the following colors: van der Waals (beige), electrostatic (blue), polar solvation (red), and non-polar solvation (grey).

**Figure 10:**
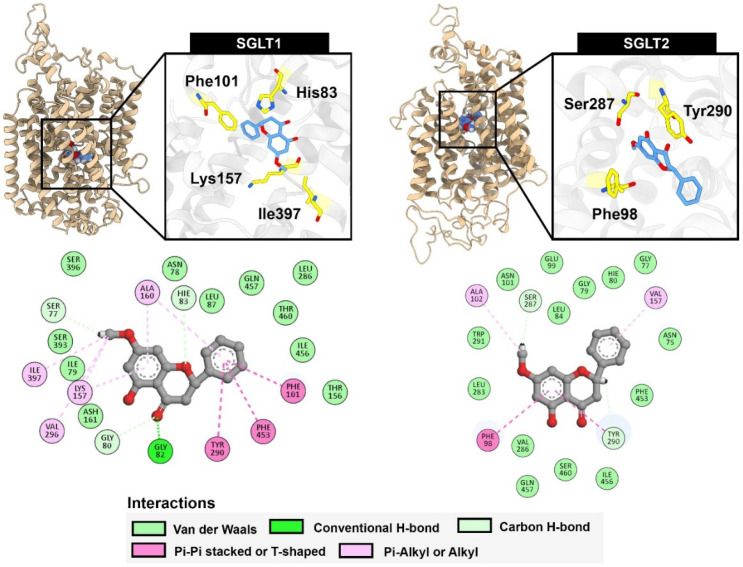
Dimensional binding poses and 2-dimensional protein-ligand interactions of pinostrobin within the substrate binding site of SGLT1 and SGLT2 extracted from the last frame of the molecular dynamics simulation trajectory.

To identify the key residues contributing to protein-ligand binding, per-residue energy decomposition analysis was performed (Figure 9 and Figure 10). In the case of SGLT1, pinostrobin favorably interacted with His83 through a hydrogen bond involving the oxygen atom of the chromanone ring, along with a π–π T-shaped interaction between the monosubstituted aromatic ring and Phe101. The presence of a methoxy group, instead of a hydroxy group, facilitated additional π–alkyl interactions with Lys157 and Ile397, which were not observed in the case of pinocembrin. For SGLT2, pinostrobin predominantly interacted with Phe98 and Tyr290 via π-π T-shaped and π-π stacked interactions, respectively. The methoxy group also contributed to a weak hydrogen bond with Ser287. These computational findings underscore the importance of the methoxy group in pinostrobin for enhancing binding interactions with SGLT1 and SGLT2.

## 3. Discussion

The isolated compounds were identified based on their ^1^H-NMR spectra, which were consistent with previously reported data for pinocembrin (C_15_H_12_O_4_) and pinostrobin (C_16_H_14_O_4_). Pinostrobin was further confirmed by its molecular mass of 270.284 Da, in agreement with literature values [15]. Structural elucidation of the two compounds, pinostrobin and pinocembrin, was unequivocally supported by MS and NMR analyses. Both compounds have previously been reported from the rhizomes of *B. rotunda*, and are recognized as major bioactive markers for the standardization of *B. rotunda* extracts [16]. The HPLC method used for quantitative analysis was validated according to the International Council for Harmonisation (ICH) guideline Q2(R1) [17], and all validation parameters met the acceptable criteria, confirming the suitability of the assay for analytical purposes. To enrich the bioactive content, solvent fractionation was employed, with the ethyl acetate fraction yielding a high concentration of chalcone derivatives. This fraction contained 21.4 ± 0.05% *w*/*w* of pinostrobin and 9.3 ± 0.02% *w*/*w* of pinocembrin. These values align with previous reports, which also demonstrated that pinostrobin tends to occur at approximately 1–2 times the concentration of pinocembrin in *B. rotunda* extracts [16]. The α-amylase inhibitory activities of pinostrobin, pinocembrin, and the crude extract of *B. rotunda* may suggest potential antidiabetic properties, as these compounds can modulate carbohydrate digestion. Among the tested substances, pinocembrin demonstrated the most highly significant pancreatic α-amylase inhibition in all tested compounds. Previous reports on the α-amylase inhibitory activity of pinocembrin have shown that racemic pinocembrin exhibits concentration-dependent inhibition, with both S- and R-enantiomers contributing additively to the overall activity [18]. This suggests that pinocembrin can effectively inhibit α-amylase activity, supporting its potential role in managing postprandial hyperglycemia. In contrast, pinostrobin and the crude extract of *B. rotunda* showed mild α-amylase inhibitory activity in the current study. However, other research has highlighted the antidiabetic potential of *B. rotunda* extracts, demonstrating that a polyphenol-rich fraction of *B. rotunda* exhibited antidiabetic properties in high fructose/streptozotocin-induced diabetic rats [19], suggesting that major bioactive compounds in the extract may contribute to its hypoglycemic effects. The observed differences in α-amylase inhibitory activities among these compounds may be attributed to their distinct chemical structures and affinities for the enzyme’s active site. Pinocembrin, a flavanone, may interact more effectively with α-amylase compared to pinostrobin, leading to higher inhibitory activity. These findings indicate that pinocembrin possesses notable α-amylase inhibitory activity, which may contribute to its antidiabetic potential. While pinostrobin and the crude extract of *B. rotunda* showed limited activity in this assay, their overall antidiabetic effects may involve additional mechanisms or other bioactive constituents, warranting further investigation to fully elucidate their comprehensive antidiabetic properties.

The in vitro α-glucosidase inhibitory activities suggest their potential as antidiabetic agents by modulating disaccharidase digestion. Pinocembrin showed the most potent α-glucosidase inhibition among the tested compounds. This finding aligns with previous studies where pinocembrin exhibited moderate α-glucosidase inhibitory activity, with IC_50_ values of 0.35 ± 0.021 mM against maltase and 0.39 ± 0.020 mM against sucrase activity [11]. The inhibition mechanism was identified through which pinocembrin interacts with both the active site and allosteric sites of the enzyme, thereby affecting both the binding affinity and the maximum reaction rate. However, pinostrobin exhibited moderate α-glucosidase inhibitory activity, though less potent than pinocembrin. Its structural similarity to pinocembrin may suggest potential for enzyme interaction, albeit with lower efficacy. The crude extract of *B*. *rotunda* showed the weakest α-glucosidase inhibitory activity among the tested samples. However, it is noteworthy that certain fractions of *B*. *rotunda* extracts not only contain an amount of specific bioactive constituents within the extract that contribute to its inhibitory effects, but they also suggest possible synergistic effects. Pinostrobin and pinocembrin inhibit maltase activity and may be influenced by specific hydroxylation patterns in their flavonoids [20]. Some studies have shown that hydroxylation at certain positions enhances inhibitory effects [21]. The structural features of pinostrobin and pinocembrin may not favor strong interactions with the enzyme’s active site, resulting in lower inhibitory activity.

Furthermore, the use of Caco-2 cells as a model for human intestinal absorption provides a relevant system for assessing the potential of compounds to inhibit α-glucosidase activity in the human intestine that closely mimics human intestinal physiology. However, it is important to consider that in vitro results may not always directly translate to in vivo efficacy due to factors such as bioavailability and metabolism. In this study, the crude extract of *B*. *rotunda* exhibits some inhibitory activity against membrane-bound α-glucosidase, and its efficacy is limited compared to established inhibitors, e.g., acarbose. The individual constituents, pinostrobin and pinocembrin, showed minimal activity, suggesting that the crude extract’s effect may result from a combination of compounds or other minor constituents. Many studies initially screen compounds for α-glucosidase inhibitory activity using the enzyme derived from *Saccharomyces cerevisiae* (yeast) due to its availability and cost-effectiveness. However, the yeast α-glucosidase differs structurally and functionally from the human intestinal membrane-bound α-glucosidase, leading to discrepancies in inhibitory efficacy observed in vitro versus in vivo. For instance, a study by Wongon and Limpeanchob demonstrated that oxyresveratrol exhibited potent inhibition of yeast α-glucosidase but showed significantly reduced efficacy against the membrane-bound α-glucosidase in differentiated Caco-2 cells [22]. Similarly, research on 9-O-berberrubine carboxylate derivatives revealed strong inhibitory activity against yeast α-glucosidase, with IC_50_ values ranging from 1.61 to 32.84 μM [23]. However, the authors emphasized the necessity of further studies involving human intestinal enzymes and cell-based experiments to validate these findings.

In the context of *B*. *rotunda*, the crude extract demonstrated a concentration-dependent inhibition of maltase activity in Caco-2 cells; the residual maltase activity was higher than pinocembrin, which exhibited minimal inhibitory effects on maltase activity at all tested concentrations. These findings suggest that while the crude extract contains components capable of inhibiting human membrane-bound α-glucosidase, the isolated flavonoids pinostrobin and pinocembrin may not be the primary active constituents responsible for this activity. This underscores the importance of evaluating compounds in human-relevant models, as results from yeast-based assays may not accurately reflect efficacy in human systems.

The observed inhibitory effects of *B*. *rotunda* extract and its constituents, pinostrobin and pinocembrin, on glucose uptake in Caco-2 cells suggest a potential in vitro model for studying intestinal glucose absorption, as it expresses key glucose transporters such as SGLT and GLUT. In the present study, the crude extract of *B*. *rotunda* significantly reduced glucose uptake in the differentiated Caco-2 model. Pinostrobin exhibited the most pronounced inhibitory effect, reducing 2-NBDG absorption. Pinocembrin also demonstrated notable inhibition, as well as the positive control, phlorizin, which showed strong inhibitory activity, reducing glucose uptake, while phloretin had a milder effect. These findings align with previous research indicating that certain flavonoids can modulate glucose transport in intestinal cells [24]. The study by Manzano and Williamson demonstrated that polyphenols and phenolic acids from strawberry and apple decreased glucose uptake and transport in human intestinal Caco-2 cells [25]. Similarly, 6-shogaol, a compound found in ginger, was shown to reduce glucose uptake in Caco-2 cells by downregulating the expression of SGLT1 and GLUT2 transporters [26]. The differential effects observed among the compounds tested may be attributed to their varying affinities for glucose transporters or their influence on transporter expression by their mechanisms of action and efficacy in vivo models. Using high-glucose environments, known inhibitors such as phlorizin (targeting SGLT) and phloretin (targeting GLUT) significantly reduced 2-NBDG transport, indicating effective inhibition of glucose transporter activity. However, the crude extract, pinostrobin, and pinocembrin did not exhibit significant effects under these conditions, suggesting limited interaction with glucose transporters when glucose is abundant. Conversely, under glucose-free conditions, pinostrobin and pinocembrin significantly reduced 2-NBDG transport, with pinostrobin at 500 μg/mL achieving a 49% reduction in absorption, comparable to phlorizin’s 45% reduction. This indicates that these compounds may modulate glucose transport mechanisms, particularly under low-glucose conditions. These findings align with previous research indicating that certain flavonoids can modulate glucose transport in intestinal cells. For instance, studies have shown that polyphenol-rich extracts can inhibit glucose uptake in Caco-2 cells by modulating the expression of glucose transporters such as GLUT2 and SGLT1 [27].

Understanding the specific interactions between these compounds and glucose transporters could provide insights into their potential as natural modulators of glucose absorption. The binding of compounds within the glucose transporter channels of SGLTs leads to the blockage of glucose from passage through the cells. We employed an in silico study to highlight the key interactions between our potential compound, pinostrobin, and the glucose transporters SGLT1 and SGLT2. The relative binding energies calculated using GNINA and MM-GBSA methods indicated the strength of binding. Lower binding energies suggested greater binding affinity, which correlates with inhibitory activity against enzymes. Compared with the positive control, phlorizin, pinostrobin showed lower binding ability in both docking scores and MM-GBSA calculations. However, pinostrobin exhibited a stronger binding ability than pinocembrin, highlighting the role of its methoxy group in interacting with amino acid residues within the binding site of SGLTs. Per-residue energy decomposition analysis revealed that the methoxy groups of pinostrobin formed various π–alkyl and alkyl interactions with key amino acids such as Lys157, Val296, and Ile397 (in the case of SGLT1), and Ala102 (in the case of SGLT2). These particular interactions were not observed with pinocembrin, suggesting that the methoxy group may represent a key pharmacophore for the development of SGLT inhibitors. In high-glucose environments, glucose transport across intestinal epithelial cells, such as Caco-2, is predominantly facilitated by facilitative glucose transporters GLUT and GLU11. GLUT2, a low-affinity, high-capacity transporter, can translocate from the basolateral to the apical membrane in response to elevated luminal glucose, enhancing glucose absorption. GLUT1, a high-affinity transporter, contributes to basal glucose uptake and remains active across varying glucose conditions. In this study, the significant reduction in 2-NBDG transport observed with phloretin treatment supports the role of GLUT2 and GLUT1 in mediating glucose transport under high-glucose conditions, as phloretin is a known inhibitor of these transporters. In contrast, pinostrobin, pinocembrin, and the crude extract of *B*. *rotunda* did not significantly inhibit glucose transport in the presence of high glucose, suggesting that their effects may not involve direct inhibition of GLUT2/GLUT1 or that their activity may be more prominent under glucose-depleted conditions. This aligns with previous findings that glucose transporter activity and localization are dynamically regulated by glucose availability [28,29]. In low-glucose environments, sodium-dependent glucose transporters (SGLT1 and SGLT2) play a critical role in active glucose uptake across the intestinal epithelium. Unlike facilitative GLUT transporters, SGLTs utilize the sodium gradient to actively transport glucose against its concentration gradient, ensuring efficient absorption when luminal glucose levels are low. SGLT1 is primarily expressed in the small intestine and is responsible for most dietary glucose uptake, while SGLT2 is mainly found in the kidneys but may have minor intestinal roles [30]. In this study, the notable reduction in 2-NBDG transport by pinostrobin, pinocembrin, and known inhibitors phlorizin and phloretin under glucose-deprived conditions suggests that these compounds may inhibit SGLT-mediated glucose uptake. Phlorizin, a well-characterized SGLT inhibitor, confirms the involvement of these transporters. This supports the hypothesis that *B*. *rotunda* compounds exert their effects predominantly by targeting SGLT function in low-glucose states, consistent with reports that active glucose transport via SGLTs is critical for maintaining glucose homeostasis when extracellular glucose is scarce [31,32]. Although our in vitro findings demonstrate that pinostrobin and pinocembrin possess promising antidiabetic activity through inhibition of glucose uptake, the clinical relevance of these results requires further investigation. Preclinical studies have demonstrated favorable safety profiles for both pinocembrin and pinostrobin. Pinocembrin exhibited no acute or sub-chronic toxicity in male Wistar rats at oral doses up to 500 mg/kg, with no mortality, significant changes in body or organ weights, or alterations in blood biochemistry [33]. Genotoxicity assessments, including liver micronucleus formation and mitotic index analysis, indicated no mutagenic effects. Additionally, pinocembrin showed organ-specific protective properties, such as hepatoprotection against carbon tetrachloride-induced liver fibrosis and neuroprotection against β-amyloid-induced neuronal damage [34]. Similarly, pinostrobin demonstrated low acute toxicity in male Wistar rats, with doses up to 500 mg/kg causing no significant adverse effects. Sub-chronic studies using fingerroot extract containing pinostrobin confirmed its safety at doses up to 100 mg/kg/day [35]. Genotoxicity evaluations revealed no mutagenic activity, and pinostrobin also exhibited renal protective effects against gentamicin-induced toxicity, likely mediated by its anti-inflammatory and antioxidant mechanisms [36]. Overall, both compounds appear to be safe in preclinical models. However, comprehensive clinical studies are necessary to confirm their safety, pharmacokinetics, and therapeutic potential in humans.

## 4. Materials and Methods

### 4.1. Plant Material and Plant Extraction

*B. rotunda* was collected in January 2024 from Srithep, Phetchabun, Thailand, and the specimen was identified by a botanist and kept in both Queen Sirikit Botanical Gardens (QSBG), Chiang Mai, Thailand, and School of Pharmaceutical Sciences, University of Phayao, Thailand, under the same code of PHARCOSUP45. Fresh rhizomes were collected. Rhizomes were collected, dried in a hot-air oven at 55 °C for 2 days, and then ground. The size of the ground powder was selected in the range of 100–250 µm and kept at reduced pressure until use. The dried plant (300 g) was macerated with 99% ethanol (2 L) and assisted by an ultrasonic bath (KQ3200DE, Kunshan Ultrasonic Instruments, Shanghai, China) at 40 kHz for 30 min. It was repeated by the new solvent three times. After that, the extract was filtered through a cellulose membrane (Whatman filter paper no. 1), (Merck, Göttingen, Germany) and the solvent was then evaporated under reduced pressure using a rotary evaporator (EYELA, N-1001, Tokyo, Japan) to obtain the crude extract (12% *w*/*w* of dried plant).

### 4.2. Isolation of Pinostrobin and Pinocembrin

The crude extract (25 g) was partitioned by hexane (1 L) and methanol (1 L) to obtain the hexane (7.5 g) and methanol fractions. The methanol fraction was then partitioned using water (500 mL) and ethyl acetate (EtOAc) (1 L) to obtain the EtOAc (17 g) and water fractions (4.2 g). The EtOAc fraction was then subjected to fractionation using a quick column chromatography (column: 10 × 13 cm) with gradient elution of hexane–EtOAc (100:0 to 0:100) followed by hexane to EtOAc (100:0 to 0:100) and then EtOAc to MeOH (100:0 to 50:50). Thirty fractions (250 mL each) were collected and named as F1-F30 (Figure 1a). The fractions 13 to 16 (P15) were pooled, evaporated, and purified by silica gel column chromatography (EtOAc–hexane, 1:9), yielding 20 fractions (P15-1 to P15-20). P15-1 to P15-8 were pooled and evaporated from the solvent and then recrystallized using DCM and MeOH (3:7) at 4 °C to provide white crystals of pinostrobin (98 mg). While P15-14 to P15-17 fractions were pooled and then recrystallized by DCM and MeOH (3:7) at 4 °C to provide yellowish powder of pinocembrin (41 mg), The isolated compounds were identified by NMR (Nuclear Magnetic Resonance Spectroscopy AVANCE NEO Bruker, (CryoProbe Prodigy) 600 MHz (Liquid)–600 MHz Prodigy NMR) (Bruker, MA, USA) and mass spectroscopy (Agilent-6540 UHD accurate-mass quadrupole-time-of-flight (QTOF) mass spectrometer (Agilent Technologies, CA, USA).

### 4.3. HPLC Method Validation for Quantification of Pinostrobin and Pinocembrin

Pinostrobin and pinocembrin were simultaneously analyzed using an HPLC-UV assay. The HPLC assay was carried out using the HPLC Shimadzu Prominence UFLC system, hyphenated with a UV-Vis detector (SPD-20A 230 V) (Shimadzu, Tokyo, Japan), and an LC-20AD pump (Shimadzu, Tokyo, Japan)was used. The sample injection volume was 20 μL, and a C-18(2) column (Phenomenex) (Santa Clara, CA, USA), 250 mm in length and 4.6 mm in diameter, was used. A flow rate of 1.50 mL/min under room temperature conditions was employed. The gradient elution was applied at different mobile phases. The mobile phase consisted of 1.0% (*v*/*v*) formic acid in water (A) and acetonitrile (B). The gradient that was eluted was as follows: 0–5 min (40% A), 5–7 min (10% A), 7–12 min (5% A) for a total analysis time of 12 min, followed by a post-run of 40% A for another 3 min. The mobile phase was filtered through a 0.45 μm filter and degassed using an ultrasonic bath before use. A UV-Vis detector was set at a wavelength of 300 nm. To ensure the reliability of the analysis assay, the HPLC method was validated based on linearity and range, lowest detection limit concentration (LOD), the lowest quantification limit concentration (LOQ), accuracy (%recovery), and precision (%RSD) following the q2(R1) ICH guideline (2005). This validated HPLC analysis was applied to standardize the bioactive compound content, pinostrobin, and pinocembrin in % *w*/*w* in the extract before bioactivity evaluation.

### 4.4. In Vitro α-Amylase Inhibition Assay

The α-amylase assay was performed following the method described by Wongon and Limpeanchob, with slight modifications [22]. The reaction product was quantified colorimetrically using reducing sugar oxidation. Briefly, 50 μL of various concentrations of *B. rotunda* extract (PE), pinostrobin, pinocembrin, or acarbose (Sigma-Aldrich, St. MO, USA) was incubated with 100 μL of starch solution (12.5 mg/mL in PBS) at 37 °C for 5 min. Subsequently, 100 μL of α-amylase solution (Sigma-Aldrich, St. MO, USA) (1.5 U/mL) was added and incubated for an additional 10 min. Then, 50 μL of the reaction mixture was transferred to react with 100 μL of 1% 3,5-dinitrosalicylic acid (DNS) reagent (Sigma-Aldrich, St. MO, USA). The mixture was heated at 95 °C for 5 min, allowed to cool, and the absorbance was measured at 540 nm.

### 4.5. In Vitro α-Glucosidase Inhibition Assay

The α-glucosidase inhibitory activity was evaluated based on the method of Wongon and Limpeanchob, with minor modifications [22]. The assay measured the release of p-nitrophenol from p-nitrophenyl-α-D-glucopyranoside (PNPG) (Sigma-Aldrich, St. MO, USA). In brief, 20 μL of α-glucosidase solution (Sigma-Aldrich, St. MO, USA) (125 mU/mL in phosphate-buffered saline, pH 6.8) was mixed with 80 μL of various concentrations of *B. rotunda* extract (PE), pinostrobin, or pinocembrin in a 96-well microplate. The mixtures were incubated at 37 °C for 10 min, followed by the addition of 100 μL of 0.2 mM PNPG. After a further incubation at 37 °C for 30 min, the absorbance was measured at 405 nm using a microplate reader (Thermo Fisher Scientific, MA, USA). Acarbose, prepared in distilled water at various concentrations, was used as the positive control.

### 4.6. Cell Culture

Caco-2 cells were obtained from the American Type Culture Collection (ATCC) and cultured in DMEM/F12 medium (Cytiva, UT, USA) supplemented with 10% fetal bovine serum (FBS) and 1% penicillin–streptomycin (Thermo Fisher Scientific, MA, USA). Cells were maintained at 37 °C in a humidified atmosphere containing 95% air and 5% CO_2_. For experiments, Caco-2 cells were seeded in 96-well plates for cell viability assays and in 24-well plates for α-glucosidase inhibition studies. Upon reaching confluence, the cells were allowed to differentiate, during which they expressed brush border enzymes such as lactase, sucrase-isomaltase, and alkaline phosphatase within 2–3 weeks, as described by Van Beers [37]. Throughout the differentiation period, the culture medium was replaced with fresh medium every two days. Fully differentiated cells (18–21 days post-seeding) were used for most experimental procedures.

### 4.7. Determination of Cell Viability

Cell viability was evaluated using the MTT assay, which is based on the conversion of MTT to an insoluble purple formazan product by mitochondrial dehydrogenases in viable cells. No such conversion occurs in non-viable (dead) cells. Caco-2 cells were treated with various concentrations of *B. rotunda* rhizome extract (PE), pinostrobin, or pinocembrin or acarbose for 24 h. Two hours before the end of the treatment period, 10 μL of MTT solution (Tokyo Chemical Industry, TK, JP) (5 mg/mL) was added to each well. Following incubation, the culture medium was removed, and the resulting formazan crystals were solubilized using a 1:1 (*v*/*v*) mixture of dimethyl sulfoxide (RCI Labscan, BKK, TH) (DMSO) and ethanol. Absorbance was measured at 595 nm using a microplate reader.

### 4.8. Membrane Bound α-Glucosidase Inhibition Assay

Membrane-bound α-glucosidase activity in Caco-2 cells was assessed with modifications based on the method described by Wongon and Limpeanchob [22]. Briefly, Caco-2 cells were seeded in 24-well plates at a density of 100,000 cells per well and cultured in DMEM/F12 medium supplemented with 10% fetal bovine serum (FBS) and 1% penicillin–streptomycin for 18–21 days to allow for differentiation. After reaching confluence, the culture medium was removed, and the cells were washed with 1 mL of PBS, followed by overnight incubation in serum- and glucose-free medium. Subsequently, 160 μL of culture medium containing *B. rotunda* extract (PE), pinostrobin, pinocembrin, or acarbose (20 μL, at various concentrations) was added and incubated for 15 min. This was followed by the addition of 20 μL of 250 mM maltose (Sigma-Aldric, HE, DEU) as the α-glucosidase substrate, and incubation continued for 4 h. Afterward, 20 μL of the culture medium was collected to determine glucose production using a glucose assay kit (Stanbio Laboratory, TX, USA.). All experiments were performed in triplicate, and α-glucosidase (maltase) inhibitory activity was expressed as percentage inhibition.

### 4.9. Glucose Uptake Assay

Caco-2 cells were seeded into black 96-well plates (Thermo Fisher Scientific Inc., MA, USA.) at a density of 20,000 cells per well and cultured for 20 days to allow for full differentiation, with media changes every three days. After the 20-day differentiation period, the cells were incubated overnight in glucose-free medium, followed by a 1 h incubation in buffer. Cells were then treated with test compounds, after which 200 μM of the fluorescent glucose analog 2-NBDG (Sigma-Aldrich, MA, USA) was added in a glucose-free medium. Following a 4 h incubation, the medium was removed, and the cells were washed three times with PBS. Intracellular fluorescence of 2-NBDG was measured using a microplate reader at an excitation wavelength of 485 nm and an emission wavelength of 528 nm. Results were expressed as the percentage of relative fluorescence intensity compared to the control group treated with 2-NBDG in the absence of test compounds.

### 4.10. Caco-2 Cell Monolayer Glucose Transport Assay

Caco-2 cell monolayers were cultured on Transwell^®^ inserts (Corning Incorporated, NY, USA.) placed in 24-well plates, following the method described by [38] with minor modifications. To assess apical-to-basolateral glucose permeability, 1 mL of serum-free, phenol red-free medium (pH 7.4, 37 °C) was added to the basolateral chamber, while 0.2 mL of the test solution containing 200 μM 2-NBDG was applied to the apical side. The experiment was conducted under both glucose-free (0 mM) and high-glucose (25 mM) conditions. After a 4 h incubation at 37 °C, samples were collected from the basolateral side. The amount of 2-NBDG transported across the cell monolayer was quantified using a fluorescence spectrophotometer at an excitation wavelength of 495 nm and an emission wavelength of 535 nm. Results were expressed as the relative amount of 2-NBDG that permeated to the basolateral compartment, with the glucose transport level of the control group set at 100%. Phloretin and phlorizin (Sigma-Aldrich, MA, USA) (2 mM) were used as positive controls.

### 4.11. Molecular Docking

The structures of macromolecules were retrieved from the RCSB Protein Data Bank, including sodium-glucose cotransporter 1 (SGLT1, PDB ID: 7WMV) [39] and sodium-glucose cotransporter 2 (SGLT2, PDB ID: 7VSI) [40]. The protein structures were prepared by removing water molecules, ions, and co-crystallized ligands. Missing residues were modeled using homology modeling via the online tool SWISS-MODEL [41]. The completed protein structures were exported in PDB format. The structures of ligands, including phlorizin, phloretin, pinostrobin, and pinocembrin, were generated using Chem3D software version 16. These structures were fully optimized using DFT calculations at the B3LYP/6-31++G (d,p) level of theory with the Gaussian16 package [42]. The optimized structures were exported in PDB format. Molecular docking experiments were conducted using GNINA docking with deep learning [43], running on Google Colab. The grid box was automatically generated based on the coordinates of the co-crystallized inhibitor, with an additional 5 Å padding in all dimensions. The exhaustiveness was set to 32 for all predictions. The results were visualized using BIOVIA Discovery Studio 2022.

### 4.12. Molecular Dynamics Simulation

Molecular dynamics (MD) simulations were conducted using the AMBER 23 package [44]. Three distinct binding poses obtained from molecular docking were selected for MD simulation. The protein–ligand complexes were prepared using the tleap module implemented in the AmberTools23 package [45]. The macromolecules were assigned standard protonation states at pH 7.4 using the online tool PDB2PQR. The protein structures were parameterized using the AMBER ff14SB force field [46]. Ligand charges were calculated at the semi-empirical AM1-BCC level of theory and parameterized using the Generalized AMBER Force Field 2 (GAFF2). Each protein–ligand complex was solvated in an octahedral box with a 12 Å spacing between the solute surface and the box edge, using TIP3P water molecules. Sodium (Na+) and chloride (Cl^−^) ions were added to neutralize the system and mimic a physiological concentration of 0.15 M. Energy minimization was performed using 5000 steps of the steepest descent algorithm followed by the conjugate gradient method. Hydrogen-involving bonds were constrained using the SHAKE algorithm. Temperature was maintained with a Langevin thermostat at a collision frequency of 2.0 ps-1, and pressure was controlled using a Berendsen barostat with a pressure relaxation time of 2.0 ps. Long-range electrostatic interactions were calculated using the particle mesh Ewald (PME) method with a cutoff of 8 Å. The system was gradually heated from 10 K to 310 K over 500 ps under the NVT ensemble. Pressure equilibration was carried out at 1 atm for 500 ps under the NPT ensemble. System equilibration was performed with positional restraints on solute atoms using force constants of 2.0, 1.0, 0.5, and 0.1 kcal/mol·Å2 for 200 ps each, followed by an unrestrained equilibration for 200 ps. Finally, production MD simulations were performed for 100 ns with three independent simulations using pmemd.cuda.SPFP, with a time step of 2 fs. Trajectories were saved every 10 ps.

### 4.13. MM-GBSA Calculation

The relative binding free energy was calculated using the Molecular Mechanics Generalized Born Surface Area (MM-GBSA) method with the MMPBSA.py script. A total of 1000 snapshots were extracted from the last 10 ns of each trajectory for the calculations. The solvation energy was computed using the modified Generalized Born model (igb = 2). The salt concentration was set to 0.15 M. The relative binding free energy was calculated using the following equation:

∆G_bind_ = ∆H − T∆S ∆H = ∆E_MM_ + ∆G_sol_
∆E_MM_ = ∆E_vdw_ + ∆E_elect_
∆G_sol_ = ∆G_polar_ + ∆G_nonpolar_

### 4.14. Statistical Analysis

The results are presented as mean ± standard error of the mean (SEM). Statistical comparisons between groups were performed using one-way analysis of variance (ANOVA), followed by Duncan’s multiple-range post hoc test to identify significant differences among means. All analyses were conducted using IBM SPSS Statistics version 22.

## 5. Conclusions

In this study, both isolated compounds, along with the standardized extract, were subsequently evaluated for their biological activities, specifically targeting carbohydrate-digesting enzymes and glucose transport in Caco-2 intestinal cells. The present study demonstrates the antidiabetic potential of *B. rotunda* and its flavonoid constituents, pinostrobin and pinocembrin, through their ability to modulate key enzymes involved in carbohydrate digestion and glucose transport in intestinal cells. Among the tested compounds, pinocembrin exhibited the most potent inhibitory activity against both pancreatic α-amylase and intestinal α-glucosidase, suggesting its role in attenuating postprandial hyperglycemia. Its inhibitory mechanism likely involves interactions at both the active and allosteric sites of α-glucosidase [11]. In contrast, pinostrobin and the crude extract of *B. rotunda* showed relatively weak enzyme inhibition, indicating that their antidiabetic effects may arise from other mechanisms or bioactive constituents within the extract. Notably, glucose uptake studies using differentiated Caco-2 cells revealed that the crude extract significantly reduced glucose absorption, with pinostrobin and pinocembrin also exhibiting inhibitory effects, particularly under glucose-depleted conditions. These findings suggest a potential mechanism involving the inhibition of sodium-dependent glucose transporters (SGLTs), rather than facilitative glucose transporters (GLUTs), which are more active in high-glucose environments. The differential activity observed under varying glucose concentrations supports the hypothesis that these compounds may exert their glucose-lowering effects more effectively under low-glucose conditions. Overall, these results highlight the potential of *B. rotunda*, especially pinocembrin, as a natural source of antidiabetic agents and underscore the importance of evaluating both isolated compounds and crude extracts in physiologically relevant models to better understand their mechanisms of action. Further in vivo investigations are needed to confirm these findings and to identify the specific bioactive constituents responsible for the observed effects.

## Figures and Tables

**Table 1:** ^1^H-NMR chemical shift value δ [ppm] for pinostrobin and pinocembrin isolated from *B*. *rotunda*.

Position	Pinostrobin	Position	Pinocembrin
	δH (J Hz)		δH (J Hz)
5-OH	12.02, s	5-OH	12.04, s
H-2′-H-6′	7.52 − 7.33, m	H-2′-H-6′	7.53 − 7.34, m
H-6, H-8	6.08, d (J = 6.1 Hz)	H-6, H-8	6.00, d (J = 1.6 Hz)
H-2	5.43, dd (J = 13.0, 2.9 Hz)	7-OH	5.66, s
7-OMe	3.82, s	H-2	5.43, dd (J = 13.0, 3.0 Hz)
H-3a	3.09, dd (J = 17.2, 13.1 Hz)	H-3a	3.09, dd (J = 17.2, 13.1 Hz)
H-3e	2.83, dd (J = 17.2, 3.0 Hz)	H-3e	2.83, dd (J = 17.2, 3.0 Hz)

**Table 2:** The results of HPLC method validation for pinocembrin and pinostrobin.

Validation Parameter	Biomarker	
	Pinostrobin	Pinocembrin
Linearity	1.00–70.00 µg/mL	1.00–70.00 µg/mL
Correlation coefficient (R^2^)	0.9999	0.9997
Linear equation	y = 27,451x + 8159.9	y = 12,391x − 7842
LOD	0.046 µg/mL	0.11 µg/mL
LOQ	0.15 µg/mL	0.30 µg/mL
Precision (%RSD); intra-day	0.12–0.62%	0.09–0.88%
Precision (%RSD); intra-day	0.69–0.75	0.33–0.70%
Accuracy (%Recovery)	108.97–109.75%	106.94–109.98%

**Table 3:** Inhibitory effects of pinostrobin and pinocembrin on glucose uptake in Caco-2 cells. Cells were pretreated with 100, 250, and 500 μg/mL of each compound for 4 h. Glucose uptake was expressed as a percentage of activity relative to the control group. Phlorizin and phloretin were used as positive controls. Data are presented as mean ± SEM from at least three independent experiments. Different symbols indicate statistically significant differences to no-compound-treated cells (* *p* < 0.05, # *p* < 0.01).

Compounds	Concentration (µg/mL)		
	100	250	500
	% Glucose Uptake		
Crude Extract	61.03 ± 8.4 *	53.81 ± 6.8 #	66.58 ± 8.2 #
Pinostrobin	77.33 ± 15.5	49.97 ± 5.0 #	50.51 ± 7.0 #
Pinocembrin	84.19 ± 5.2	64.11 ± 8.4 *	55.01 ± 9.2 *
Phloretin	76.81 ± 14.7	69.70 ± 16.3	64.43 ± 12.2 *
Phlorizin	70.90 ± 9.7 *	70.79 ± 6.1 #	56.33 ± 6.8 #

**Table 4:** Relative binding energy between potent inhibitors and SGLT1 and SGLT2 receptors calculated from molecular docking and molecular dynamics simulations.

Compound	Relative Binding Free Energy (kcal/mol)	
	GNINA	MM-GBSA
**SGLT1**		
phlorizin	−11.36	−67.71 ± 4.29
phloretin	−9.81	−47.20 ± 3.77
pinostrobin	−7.43	−40.31 ± 3.21
pinocembrin	−7.82	−30.47 ± 2.47
**SGLT2**		
phlorizin	−12.46	−68.68 ± 4.87
phloretin	−9.12	−34.18 ± 3.52
pinostrobin	−8.38	−34.18 ± 3.52
pinocembrin	−5.55	−30.50 ± 2.59

## Data Availability

The data that support the findings of this study are available from the corresponding author upon reasonable request.

## Supplementary Materials

The following supporting information can be downloaded at: https://www.mdpi.com/article/10.3390/ijms262010158/s1.
