# Supplementary material for: Inhibitory Effect of Boesenbergia rotunda and Its Major Flavonoids, Pinostrobin and Pinocembrin on Carbohydrate Digestive Enzymes and Intestinal Glucose Transport in Caco-2 Cells
Source: Int J Mol Sci. 2025 Oct 19;26(20):10158. doi: 10.3390/ijms262010158 (PMC12562543; doi:10.3390/ijms262010158)
Supplement: Supplementary file 1 [file ijms-26-10158-s001.zip › ijms-3899756-supplementary.pdf]

Supporting Information

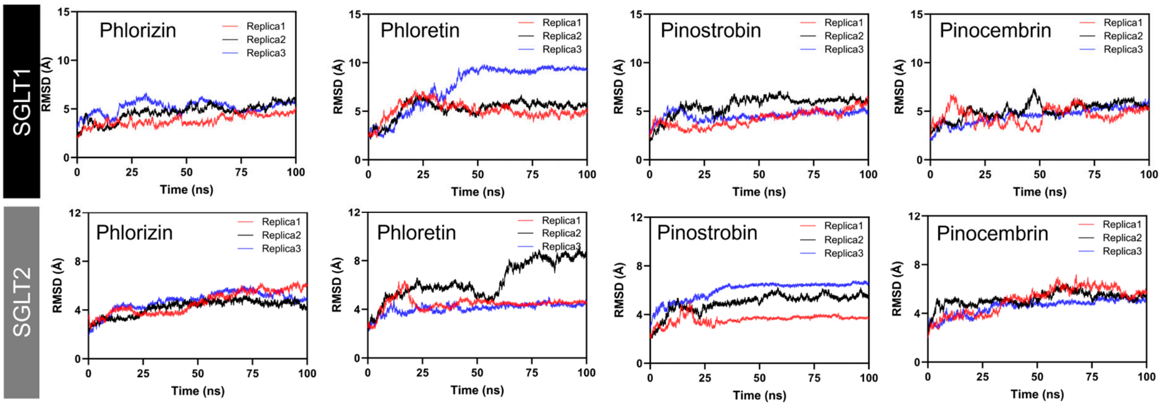

Figure S1. Root-mean-square-deviation (RMSD) of the Cα of protein structures of three independent molecular dynamics simulations.

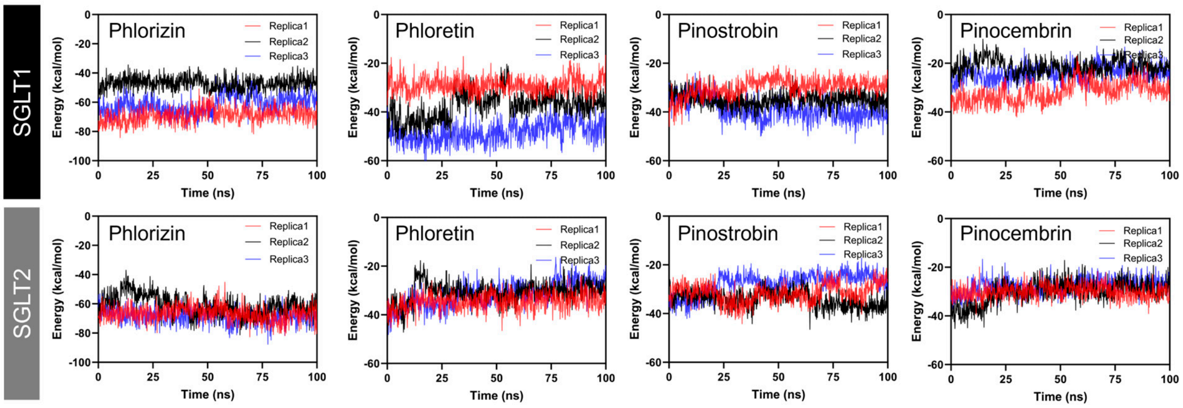

Figure S2. Evolution of binding energy over the time of three independent molecular dynamics simulations of potent compounds with SGLT1, SGLT2, and GLUT1.
